# PM2.5 induces lung injury via mtDNA-cGAS-STING-mediated macrophage M1 polarization

**DOI:** 10.3389/fphar.2026.1902928

**Published:** 2026-08-06

**Authors:** Bingbing Yan, Bomiao Qing, Manling Jiang, Qin Ran, Anying Xiong, Xiang He, Junyi Wang, Lei Zhang, Keyue Wang, Xiaolan Li, Guoping Li

**Affiliations:** 1 Clinical Medicine Department, North Sichuan Medical College, Nanchong, China; 2 Laboratory of Allergy and Precision Medicine, Chengdu Institute of Respiratory Health, Affiliated Hospital of Southwest Jiaotong University, The Third People’s Hospital of Chengdu, Chengdu, China; 3 Department of Pulmonary and Critical Care Medicine, Chengdu Third People’s Hospital Branch of National Clinical Research Center for Respiratory Disease, Affiliated Hospital of Chongqing Medical University, Chengdu, China

**Keywords:** cGAS-STING, lung injury, macrophage polarization, mtDNA, PM2.5

## Abstract

**Background:**

Exposure to fine particulate matter (PM2.5) is a well-established risk factor for lung inflammation and injury. Macrophages are key innate immune cells in the lung and play critical roles in maintaining pulmonary immune homeostasis and orchestrating inflammatory responses following environmental insults. However, the precise mechanisms by which PM2.5 modulates macrophage function and contributes to lung injury remain incompletely understood. This study aimed to investigate the role of macrophage phenotypic polarization in PM2.5-induced lung injury and the underlying molecular mechanisms.

**Methods:**

A subacute exposure (21-day) PM2.5 mouse model and bone marrow-derived macrophages (BMDMs) were used *in vivo* and *in vitro* to evaluate the effects of PM2.5 on pulmonary inflammation, macrophage polarization, mitochondrial injury, and cGAS-STING signaling activation. Pharmacological inhibition of cGAS was performed using RU.521 to assess the functional role of the cGAS-STING pathway in PM2.5-induced macrophage activation and lung injury. Statistical analyses were conducted to compare differences between experimental groups.

**Results:**

PM2.5 exposure triggered pulmonary inflammation and tissue injury, accompanied by increased pulmonary macrophage accumulation and polarization toward the pro-inflammatory M1 phenotype. Mechanistically, PM2.5 induced mitochondrial damage in macrophages, leading to mtDNA release and subsequent activation of cGAS-STING signaling. Pharmacological inhibition of cGAS with RU.521 attenuated STING pathway activation, macrophage M1 polarization, and PM2.5-induced pulmonary inflammation and lung injury in mice.

**Conclusions:**

Our study indicates that PM2.5 promotes lung injury by driving macrophage M1 polarization through an mtDNA-cGAS-STING axis, highlighting cGAS-STING signaling as a potential therapeutic target for PM2.5-related lung injury.

## 1 Introduction

Fine particulate matter (PM2.5, aerodynamic diameter ≤2.5 μm), one of the most prevalent environmental pollutants, has been widely recognized as a major risk factor for the global burden of disease. Owing to its small size and large surface area, PM2.5 can penetrate deep into the respiratory tract and deposit in the alveoli, thereby exerting sustained biological effects on lung tissue (Piao et al., 2023). Accumulating evidence has indicated that PM2.5 exposure contributes to multiple respiratory diseases, including asthma, chronic obstructive pulmonary disease (COPD), and pulmonary fibrosis (Zhao et al., 2020). Despite their distinct clinical manifestations and pathological characteristics, many respiratory diseases share common pathological processes, particularly pulmonary inflammation and structural remodeling, that are closely associated with PM2.5-induced lung injury. Therefore, understanding the mechanisms of PM2.5-induced lung injury is essential for developing effective preventive and therapeutic strategies.

Pulmonary macrophages represent a predominant innate immune cell population in the lung, serving as pivotal mediators of immune homeostasis and initiating responses to inhaled particulate and environmental challenges. Under physiological conditions, pulmonary macrophages limited excessive inflammation by phagocytosing inhaled particles and pathogens and clearing apoptotic cells, thereby maintaining a relatively immunosuppressive microenvironment (Malainou et al., 2023). However, under sustained stimulation, macrophages underwent functional reprogramming toward a classically activated (M1) phenotype, characterized by increased production of pro-inflammatory cytokines and mediators (Wu et al., 2024). M1-polarized macrophages have been implicated in the pathogenesis of multiple lung diseases, including acute lung injury, chronic obstructive pulmonary disease, and asthma (Jiao et al., 2021; Feng et al., 2023; Britt et al., 2023). Notably, PM2.5 exposure markedly altered the phenotype and function of pulmonary macrophages. Previous studies showed that PM2.5 directly activated macrophages, promoted M1 polarization, and induced sustained release of pro-inflammatory cytokines such as Tnf-α, Il-1β, and Il-6, thereby exacerbating pulmonary inflammation (Lee et al., 2023). In addition, PM2.5-stimulated macrophages secreted various chemokines that facilitated the recruitment of neutrophils and monocytes into lung tissue, further amplifying inflammatory cascades and aggravating lung injury (Lin et al., 2022). Nevertheless, the molecular mechanisms governing PM2.5-induced M1 polarization of pulmonary macrophages and their contribution to PM2.5-related lung injury remain to be fully elucidated.

The cGAS-STING signaling pathway is an important cytoplasmic DNA sensing pathway that regulates innate immune responses and inflammatory reactions (Wu et al., 2013). Studies have shown that under cellular stress conditions such as DNA damage, mitochondrial dysfunction, and oxidative stress, the accumulation of cytosolic DNA activated the cGAS-STING signaling and subsequently upregulated the expression of downstream inflammation-related genes (Kim et al., 2023). Emerging evidence suggested that the cGAS-STING pathway contributes to macrophage polarization, with sustained activation favoring M1-like pro-inflammatory responses, whereas pharmacological inhibition of this pathway may attenuate inflammation and promote macrophage phenotypic reprogramming (Shao et al., 2024). In particular, RU.521, a selective cGAS inhibitor, has been reported to suppress cGAS-mediated inflammatory signaling in macrophages and alleviate cGAS-STING-dependent pro-inflammatory macrophage polarization (Rui et al., 2024; Jiao et al., 2025). Notably, PM2.5 exposure has been reported to induce mitochondrial dysfunction, oxidative stress, and DNA damage in lung tissue (Liu et al., 2023). These observations suggest that PM2.5 exposure may provide a favorable cellular stress environment for the activation of the cGAS-STING signaling pathway. However, the role of cGAS-STING in PM2.5-induced macrophage M1 polarization and its contribution to lung injury remains unclear.

In this study, we established both *in vivo* and *in vitro* PM2.5 subacute exposure models to investigate the molecular mechanisms underlying PM2.5-induced lung injury. Specifically, we focused on whether PM2.5-induced mitochondrial damage triggers mtDNA release and subsequently activates cGAS-STING signaling, which promoting macrophage M1 polarization and inflammatory lung injury. Our findings could provide a new insight into the mtDNA-cGAS-STING axis in PM2.5-induced lung injury and highlights potential therapeutic targets for intervention.

## 2 Materials and methods

### 2.1 PM2.5 collection and extraction

The collection and extraction of PM2.5 were conducted as previously described (Li et al., 2024; He et al., 2021). Briefly, ambient PM2.5 was collected at a fixed urban site using a high-volume air sampler installed on the rooftop of Chengdu Third People’s Hospital, a central urban location characterized by dense residential areas and heavy traffic. The quartz fiber filters containing deposited PM2.5 were cut into 1 cm × 1 cm sections and subjected to ultrasonic agitation in ultrapure water to obtain the water-soluble fraction of ambient PM2.5. The aqueous extracts were subsequently lyophilized, reconstituted in sterile PBS, autoclaved, and stored at −80 °C until further use. To minimize particle aggregation, PM2.5 suspensions were sonicated for 30 min immediately prior to experimentation.

### 2.2 Animal treatment

Female and male C57BL/6 mice, approximately 6–8 weeks old were purchased from Chengdu Dossy Experimental Animals Co., Ltd., (Chengdu, China). All animal experiments were conducted in accordance with the institutional guidelines for the care and use of laboratory animals and were approved by the Animal Ethics Committee of Southwest Jiaotong University. Briefly, after a 1-week acclimatization period, mice were randomly assigned to two groups. Animals were administered PM2.5 (50 μL) by intranasal instillation at a dose of 5 mg/kg body weight or an equivalent volume of PBS for 21 consecutive days. Based on an average mouse body weight of approximately 20 g, the corresponding daily dose was calculated as follows: 5 mg/kg × 0.02 kg = 0.10 mg, equivalent to 100 μg of PM2.5 per mouse per day.

The PM2.5 dose was selected based on our previous study and related PM2.5 exposure models (Liu et al., 2025; Liu et al., 2022; Ding et al., 2024), in which comparable doses induced reproducible pulmonary inflammation and lung injury. In addition, based on the Grade II limit of the Chinese Ambient Air Quality Standard, which defines the 24 h average PM2.5 concentration limit as 0.075 mg/m^3^, the estimated daily pulmonary exposure dose for mice is approximately 10 μg/day. Accordingly, the dose used in this study, 100 μg of PM2.5 per mouse per day, was approximately ten-fold higher than this environmental reference dose and was applied to establish a reproducible 21-day subacute PM2.5 exposure model for mechanistic investigation.

For the *in vivo* experiments involving RU.521 treatment, mice were randomly divided into four groups: Control, PM2.5, RU.521, and PM2.5 + RU.521. RU.521 (HY-114180, MCE) was first dissolved in DMSO and then diluted in a vehicle solution consisting of 10% DMSO, 40% PEG300, 5% Tween-80, and 45% saline. Mice in the PM2.5 + RU.521 group received RU.521 at a dose of 5 mg/kg by intraperitoneal injection every other day during the PM2.5 exposure period. Mice in the RU.521 group received RU.521 alone using the same dose, route, and schedule. Vehicle-treated mice received an equivalent volume of the vehicle solution by intraperitoneal injection according to the same schedule. PM2.5 was administered by intranasal instillation as described above.

### 2.3 Primary BMDM cell culture and treatment

BMDMs were prepared following a well-established protocol (Toda et al., 2021). Briefly, bone marrow cells were harvested from the femurs and tibias of 6–8-week-old male and female wild-type C57BL/6J mice. Following red blood cell lysis, the bone marrow cells were differentiated into macrophages by culturing in DMEM medium supplemented with 15% FBS and 20 ng/mL recombinant mouse macrophage colony-stimulating factor (315-02, Peprotech) for 7 days. Subsequently, the cells were stained with APC-conjugated anti-mouse CD11b antibody (17-0112-81, Invitrogen) and FITC-conjugated anti-mouse F4/80 antibody (123108, BioLegend) for 30 min at 4 °C. Flow cytometric analysis confirmed that the proportion of CD11b^+^ F4/80^+^ cells exceeded 90%. BMDMs were then used for subsequent experiments.

For the *in vitro* experiments involving RU.521 treatment, BMDMs were divided into four groups: Control, PM2.5, RU.521, and PM2.5 + RU.521. RU.521 was dissolved in DMSO to prepare a stock solution. BMDMs in the PM2.5 + RU.521 group were co-treated with PM2.5 (150 μg/mL) and RU.521 at a final concentration of 2 μM for 24 h (Vincent et al., 2017). BMDMs in the RU.521 group were treated with RU.521 alone at the same concentration and duration. An equivalent volume of DMSO was added to the solvent control group, and the final concentration of DMSO was maintained below 0.1% in all culture systems.

### 2.4 Western blot

Total protein was extracted using RIPA lysis buffer (Beyotime, China) supplemented with 1 mM PMSF (Beyotime, China) and a protein phosphatase inhibitor (Solarbio, China). Equal amounts of protein were resolved by SDS-PAGE (Vazyme, China) and transferred onto polyvinylidene fluoride (PVDF) membranes (Millipore, USA). The membranes were blocked with 5% non-fat milk at room temperature for 1 h and then incubated with primary antibodies overnight at 4 °C. After three washes with TBST, the membranes were incubated with secondary antibodies at room temperature for 2 h. Immunoreactive bands were visualized using the eBlot Touch Imager and quantified with ImageJ software. Primary antibodies used in this study: anti-cGAS (31659S, CST); anti-TMEM173/STING (19851-1-AP, Proteintech); anti-TBK1 (sc-52957, Santa cruz); anti-IRF3 (11312-1-AP, Proteintech); anti-NF-κB (8242S, CST); anti-p-NF-κB (3033S, CST); anti-p-TBK1 (5483T, CST). Secondary antibodies used in this study: anti-rabbit IgG (7074S, CST); anti-mouse IgG (AB205719, Abcam).

### 2.5 Quantitative real-time PCR

The total RNA was extracted using Trizol reagent (Vazyme, China) and reverse-transcribed into cDNA using HiScript II Reverse Transcriptase (Vazyme, China) according to the manufacturer’s instructions. Quantitative real-time PCR (qRT-PCR) was conducted on a Real-Time PCR System using SYBR Green PCR Master Mix (Vazyme, China). Relative mRNA expression levels were determined using the 2^^−ΔΔCT^ method and normalized to GAPDH. The primer sequences used are provided in Supplementary Table S1.

### 2.6 H&E, Masson and PAS staining

Lung tissues were fixed in 4% paraformaldehyde (Biosharp, China) for 48 h, dehydrated, embedded in paraffin, and sectioned at 5 μm thickness. H&E (Solarbio, China), Masson (Solarbio, China) and PAS staining (Solarbio, China) were performed according to the manufacturer’s instructions. Section images were captured using an automatic multispectral scanning microscopy system (Olympus, Tokyo, Japan) and subsequently analyzed with Image J.

### 2.7 Immunofluorescence staining

Lung tissue sections were deparaffinized, subjected to antigen retrieval, and allowed to cool to room temperature. The sections were then permeabilized with 0.3% Triton X-100 for 15 min and blocked with blocking buffer at room temperature for 30 min. Subsequently, the sections were incubated with primary antibodies overnight at 4 °C. After three washes with PBS, the sections were incubated with fluorophore-conjugated secondary antibodies for 1 h at room temperature in the dark. Nuclei were counterstained with DAPI (Biosharp, China) for 5 min. Finally, images were captured using an automatic multispectral scanning microscopy system (Olympus, Tokyo, Japan) and analyzed with ImageJ software. The secondary antibody used in this study: anti-rabbit IgG Fab2 Alexa Fluor(R) 647 Molecular Probes (4414S, CST), anti-rabbit IgG Fab2 Alexa Fluor(R) 555 Molecular Probes (4413S, CST).

### 2.8 Flow cytometry

BMDMs were seeded in 6-well plates. After 24 h of exposure to PM2.5 (150 μg/mL), cells were collected and resuspended in PBS supplemented with 2% FBS. Cells were then incubated with anti-Mo F4/80 (2608838, Thermofisher), anti-mouse CD86 (105012, Biolegend) and anti-Mo CD206 (2848388,Thermofisher) for 30 min at 4 °C in the dark. After three washes with PBS, the cells were resuspended in PBS and analyzed using a flow cytometer (Sony). Data acquisition and analysis were performed with FlowJo software.

### 2.9 Measurement of mitochondrial membrane potential

TMRE is a cell-permeable, orange-red cationic fluorescent probe that selectively accumulates in mitochondria with intact membrane potential, whereas mitochondrial depolarization or dysfunction leads to reduced TMRE retention. BMDMs were seeded into 6-well plates and exposed to PM2.5 (150 μg/mL) for 24 h. Following treatment, the cells were incubated with TMRE working solution at 37 °C for 30 min and subsequently analyzed by flow cytometry. Data were processed and quantified using FlowJo software.

### 2.10 Measurement of ROS levels

BMDMs were seeded into 6-well plates and exposed to PM2.5 (150 μg/mL) for 24 h. Following treatment, the cells were incubated with ROS-sensitive probe 2′,7′-dichlorodihydrofluorescein diacetate (H_2_DCFDA), at 37 °C for 30 min and subsequently analyzed by flow cytometry. Data were processed and quantified using FlowJo software.

### 2.11 DNA isolation and mtDNA copy number assay

Cytosolic DNA was extracted using a previously described method (Bryant et al., 2022). Briefly, BMDMs were treated with PM2.5 for 24 h, harvested, and lysed with digitonin-containing buffer to release cytosolic DNA. The supernatant was collected, and DNA was purified using a micro-DNA extraction kit (D6296-01, Omega). DNA concentration was determined with a Qubit fluorometer (ThermoFisher Scientific, USA). qRT-PCR was performed to evaluate mtDNA levels. The mtDNA copy number was normalized to that of the nuclear ribosomal protein S18.

### 2.12 BALF collection and total cell counting

After mice were sacrificed, the trachea was exposed and cannulated with a fine catheter. The lungs were lavaged three times with 0.8 mL pre-cooled sterile phosphate-buffered saline (PBS), and all recovered BALF was pooled and placed on ice. The collected BALF was centrifuged at 300 × *g* for 10 min at 4 °C to precipitate total inflammatory cells. The cell pellet was resuspended in 100 μL PBS, and total cell number was counted using a hemocytometer under an optical microscope.

### 2.13 Statistical analysis

All data are presented as the mean ± standard error of the mean (SEM). Statistical analyses were performed using GraphPad Prism version 8.0. Comparisons between two groups were assessed using an unpaired Student’s t-test. For comparisons among more than two groups, one-way or two-way analysis of variance (ANOVA) was performed, as appropriate, followed by Tukey’s multiple comparisons test. A p value < 0.05 was considered statistically significant.

## 3 Results

### 3.1 PM2.5 subacute exposure induces lung injury in mice

**FIGURE 1:**
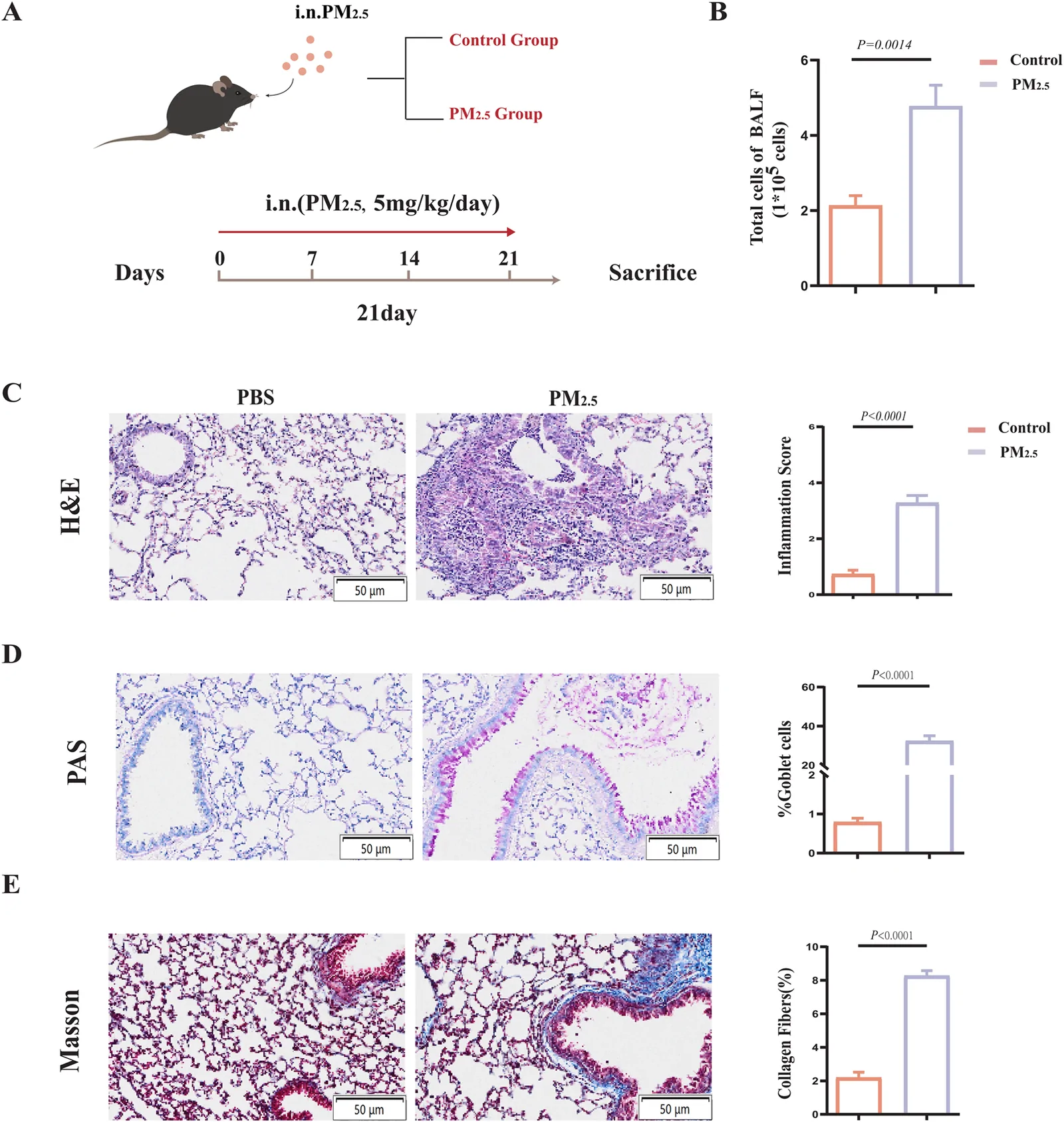
PM2.5 subacute exposure induced lung injury. **(A)** Schematic diagram of the establishment of the PM2.5- subacute exposed mice model. **(B)** The total cell counts in BALF of mice. **(C)** The representative H&E -stained images of lung tissues (left) and inflammatory area scores (right). **(D)** The representative PAS-stained images of lung tissues (left) and quantitative analysis of the percentage of PAS-positive goblet cells (right). **(E)** Masson’s trichrome staining (left) and quantitative analysis of the collagen-positive area (right). Data are shown as mean ± SEM, n = 3.

To investigate the impact of PM2.5 exposure on lung injury, we established a mouse model of PM2.5 subacute exposure by intranasal instillation for 21 days (Figure 1A). Compared with PBS-treated controls, mice exposed to PM2.5 exhibited a significant increase in the total cell counts in bronchoalveolar lavage fluid (BALF) (Figure 1B). H&E staining exhibited heightened infiltration of inflammatory cells around the airways in PM2.5-exposed mice, accompanied by higher inflammatory scores relative to controls (Figure 1C). PAS staining showed increased mucus secretion within the airway epithelium of PM2.5-exposed mice (Figure 1D). In addition, Masson staining further demonstrated enhanced collagen deposition in the lung following PM2.5 exposure (Figure 1E). These results indicate that PM2.5 exposure induced pulmonary inflammation and pathological changes characterized by collagen deposition and mucus hypersecretion.

### 3.2 PM2.5 subacute exposure induced pulmonary macrophage accumulation and M1 polarization

**FIGURE 2:**
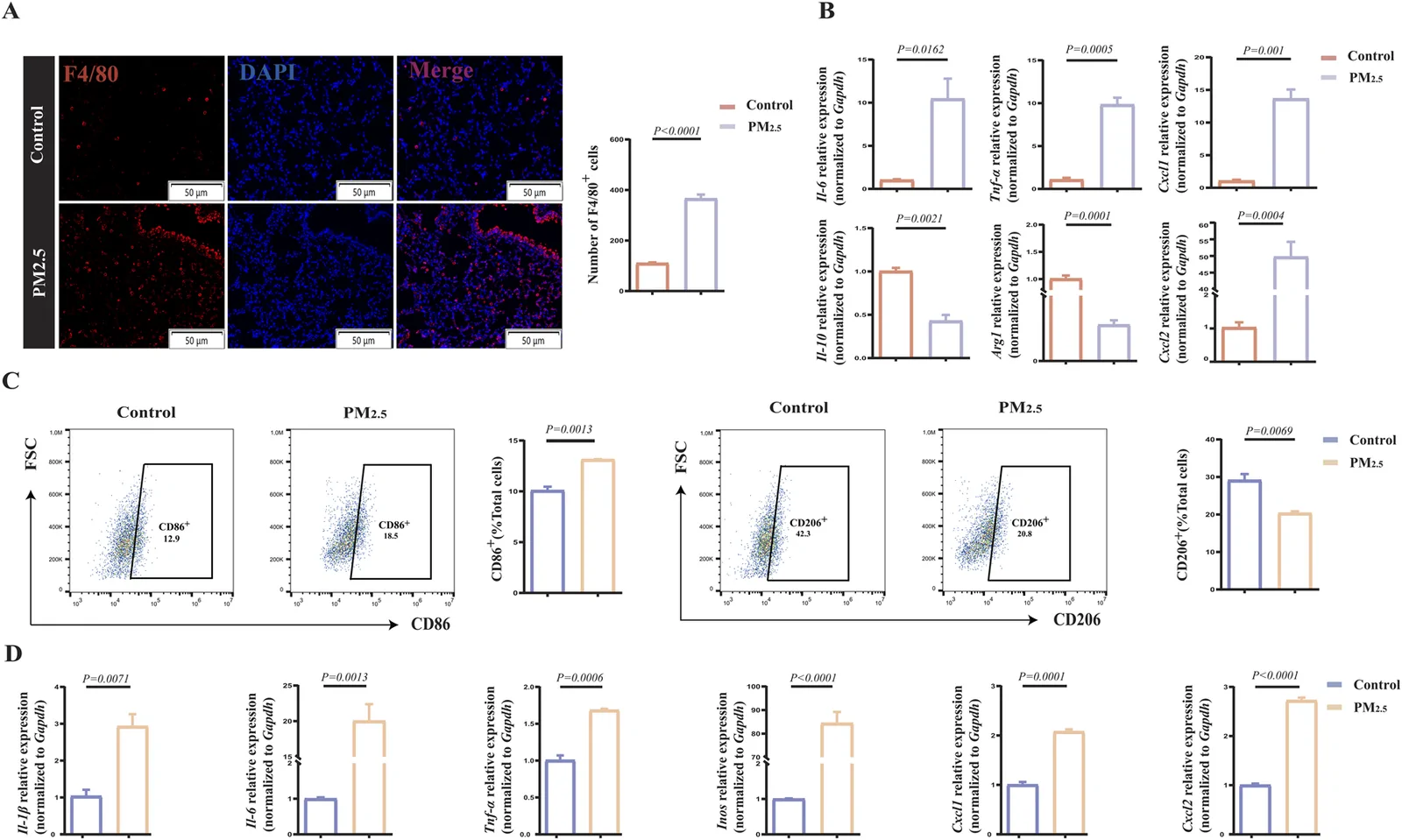
PM2.5 promoted pulmonary macrophage accumulation and polarization toward the M1 phenotype. **(A)** Representative immunofluorescence images (left) and quantitative analysis (right) of F4/80-positive pulmonary macrophages in lung tissues. **(B)** qRT-PCR analysis of the mRNA levels of inflammatory factors (*Il-6, Tnf-α, Il-10, Arg1*) and chemokines (*Cxcl1, Cxcl2*) in lung tissues of each group of mice. **(C)** Flow cytometry analysis of the proportion of CD86-positive and CD206-positive BMDMs. **(D)** Detection of expression levels of inflammatory factors (*Il-1β, Il-6, Tnf-α, iNOS*) and chemokines (*Cxcl1, Cxcl2*) in BMDMs of each group by qRT-PCR. Data are shown as mean ± SEM, n = 3.

Macrophages are widely recognized as critical mediators of pulmonary inflammatory responses (Xiong et al., 2021; Shen et al., 2023). Therefore, we next examined the effects of PM2.5 subacute exposure on pulmonary macrophages. Compared with controls, PM2.5 exposure resulted in a marked increase in F4/80-positive cells in lung tissues, indicating enhanced macrophage accumulation (Figure 2A) Consistently, qRT-PCR showed that the mRNA expression of M1 phenotype–associated pro-inflammatory cytokines *Il-6* and *Tnf-α*, as well as the chemokines *Cxcl1* and *Cxcl2*, were significantly increased in the lungs of PM2.5-exposed mice, whereas the expression of M2 phenotype-associated markers, including *Arg1* and *Il-10*, was significantly decreased (Figure 2B). To further validate these *in vitro*, primary BMDMs were isolated and exposed to PM2.5. Flow cytometric analysis showed that PM2.5 treatment significantly increased the proportion of BMDMs positive for the M1 marker CD86, while markedly decreasing the proportion of cells positive for the M2 marker CD206 (Figure 2C). In parallel, qRT-PCR analysis revealed that PM2.5 exposure significantly upregulated the expression of pro-inflammatory cytokines, including *Il-1β*, *Il-6*, *Tnf-α*, and *iNOS*, as well as the chemokines *Cxcl1* and *Cxcl2*, in BMDMs (Figure 2D). Collectively, these findings indicate that PM2.5 promoted pulmonary macrophage accumulation and drived macrophage polarization toward a pro-inflammatory M1 phenotype, thereby potentially exacerbating pulmonary inflammation.

### 3.3 PM2.5 induces cGAS-STING pathway activation in macrophage

**FIGURE 3:**
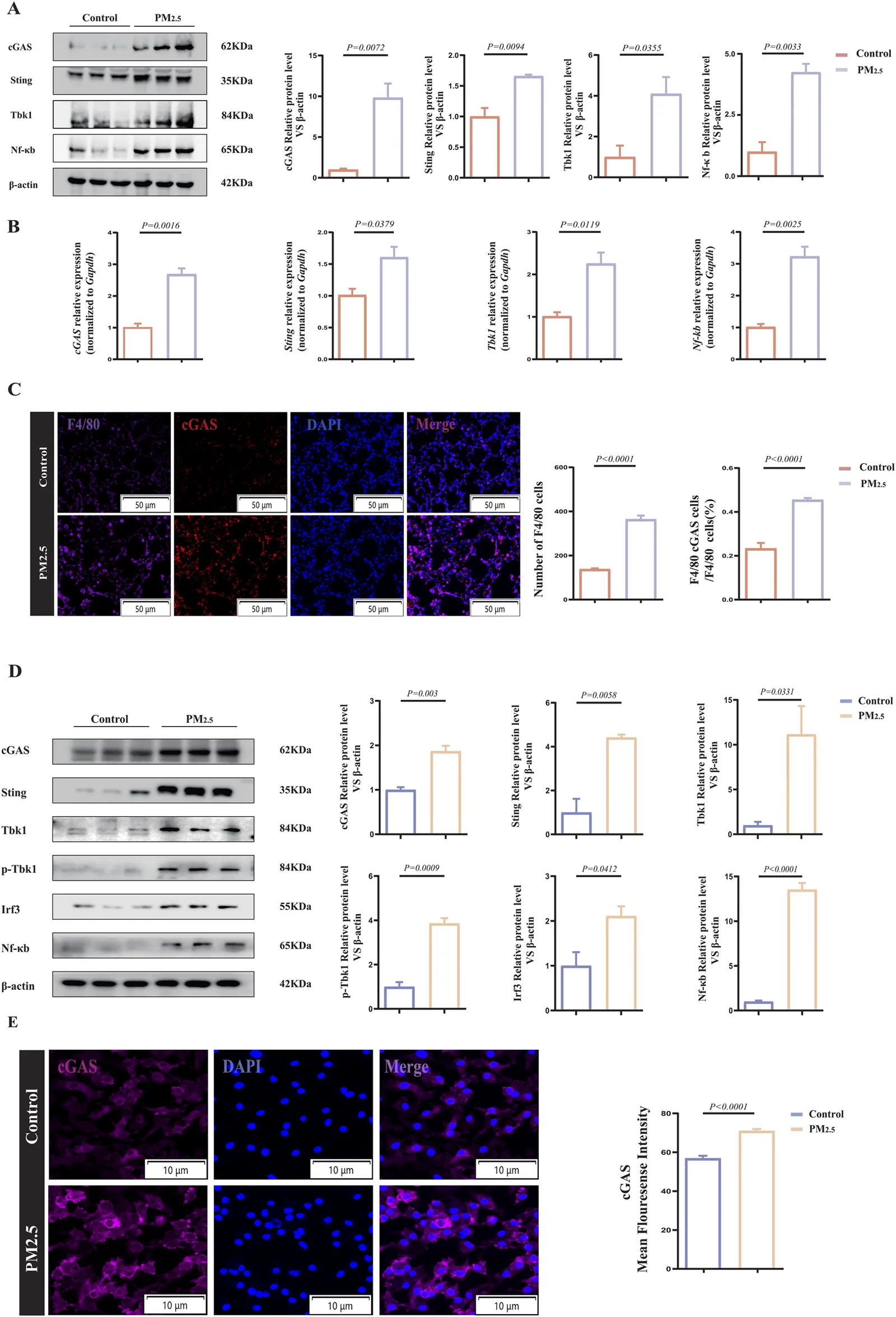
PM2.5 subacute exposure activated the cGAS-STING pathway. **(A)** Detect the expression levels of key proteins in the cGAS-STING pathway in mouse lung tissue by Western blot. **(B)** Detect the expression levels of key genes in the cGAS-STING pathway in lung tissue by qRT-PCR. **(C)** Immunofluorescence analysis of the macrophage marker F4/80 (purple) and cGAS (red) expression and co-localization in lung tissues from each group of mice, with DAPI (blue) nuclear counterstain. **(D)** Detect the expression levels of key proteins in the cGAS-STING pathway in BMDMs by Western blot; **(E)** Immunofluorescence to detect the expression of cGAS (purple) in BMDM. Data are shown as mean ± SEM, n = 3.

Accumulating evidence has established a pivotal role for cGAS-STING signaling in driving macrophage M1 polarization in response to cellular stress and inflammatory stimuli (Shen et al., 2023). We subsequently evaluated the activation of the cGAS-STING signaling pathway in macrophages following PM2.5 subacute exposure. Compared with the control group, the protein levels of cGAS, STING, TBK1 and NF-κB were increased in the lung tissue of PM2.5-exposed mice (Figure 3A). qRT-PCR results showed that the mRNA expression levels of these key genes were also significantly increased (Figure 3B). Immunofluorescence staining further confirmed that the expression level of cGAS in macrophages was markedly elevated in PM2.5-exposed lung tissue (Figure 3C). Similarly, Western blot showed that the expression levels of key proteins of the cGAS-STING pathway (cGAS, STING, TBK1, p-TBK1, IRF3, and NF-κB) in BMDMs were significantly upregulated after PM2.5 exposure (Figure 3D). Immunofluorescence analysis suggested enhanced cGAS expression in BMDMs after exposure to PM2.5 (Figure 3E). These results suggest that PM2.5 subacute exposure activated the cGAS-STING signaling pathway in macrophages both *in vivo* and *in vitro*.

### 3.4 cGAS inhibition by RU.521 attenuates PM2.5-induced M1 polarization of macrophages

**FIGURE 4:**
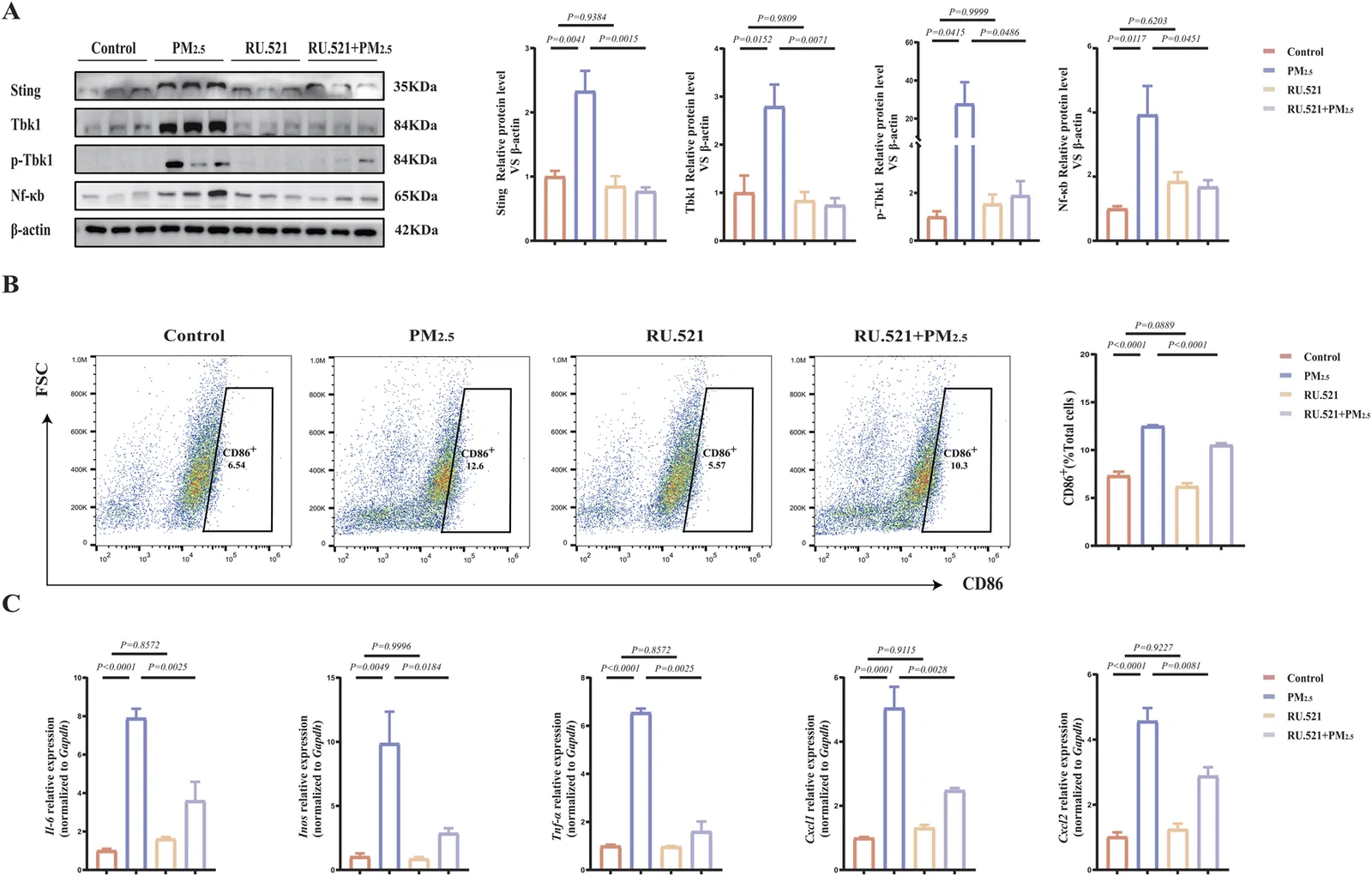
RU.521 attenuated PM2.5-induced macrophage M1 polarization. **(A)** Western blot analysis of STING, TBK1, p-TBK1 and NF-κB protein levels in different groups. **(B)** The proportion of CD86-positive BMDMs was detected by flow cytometry. **(C)** qRT-PCR detecting the expression levels of *Il-6, Tnf-α, iNOS, Cxcl1* and *Cxcl2* in control and PM2.5-treated BMDMs. Data are shown as mean ± SEM, n = 3.

To further determine whether cGAS-STING signaling mediates PM2.5-induced macrophage M1 polarization, BMDMs were co-treated with the cGAS-specific inhibitor RU.521. Western blot showed that RU.521 markedly suppressed the PM2.5-induced upregulation of STING, TBK1, p-TBK1, and NF-κB in BMDMs, indicating effective inhibition of STING pathway activation (Figure 4A). Flow cytometric analysis further demonstrated that RU.521 significantly reduced the PM2.5-induced increase in the proportion of CD86-positive macrophages (Figure 4B). Consistently, qRT-PCR results showed that the upregulation of pro-inflammatory factor genes (*Il-6, Tnf-α, iNOS*), as well as chemokines *Cxcl1* and *Cxcl2* induced by PM2.5 were inhibited after RU.521 treatment (Figure 4C). Collectively, these results indicate that pharmacological inhibition of cGAS effectively suppressed PM2.5-induced activation of the STING pathway and attenuated macrophage M1 polarization.

### 3.5 PM2.5-induced mtDNA release contribute to cGAS-STING activation in macrophages

**FIGURE 5:**
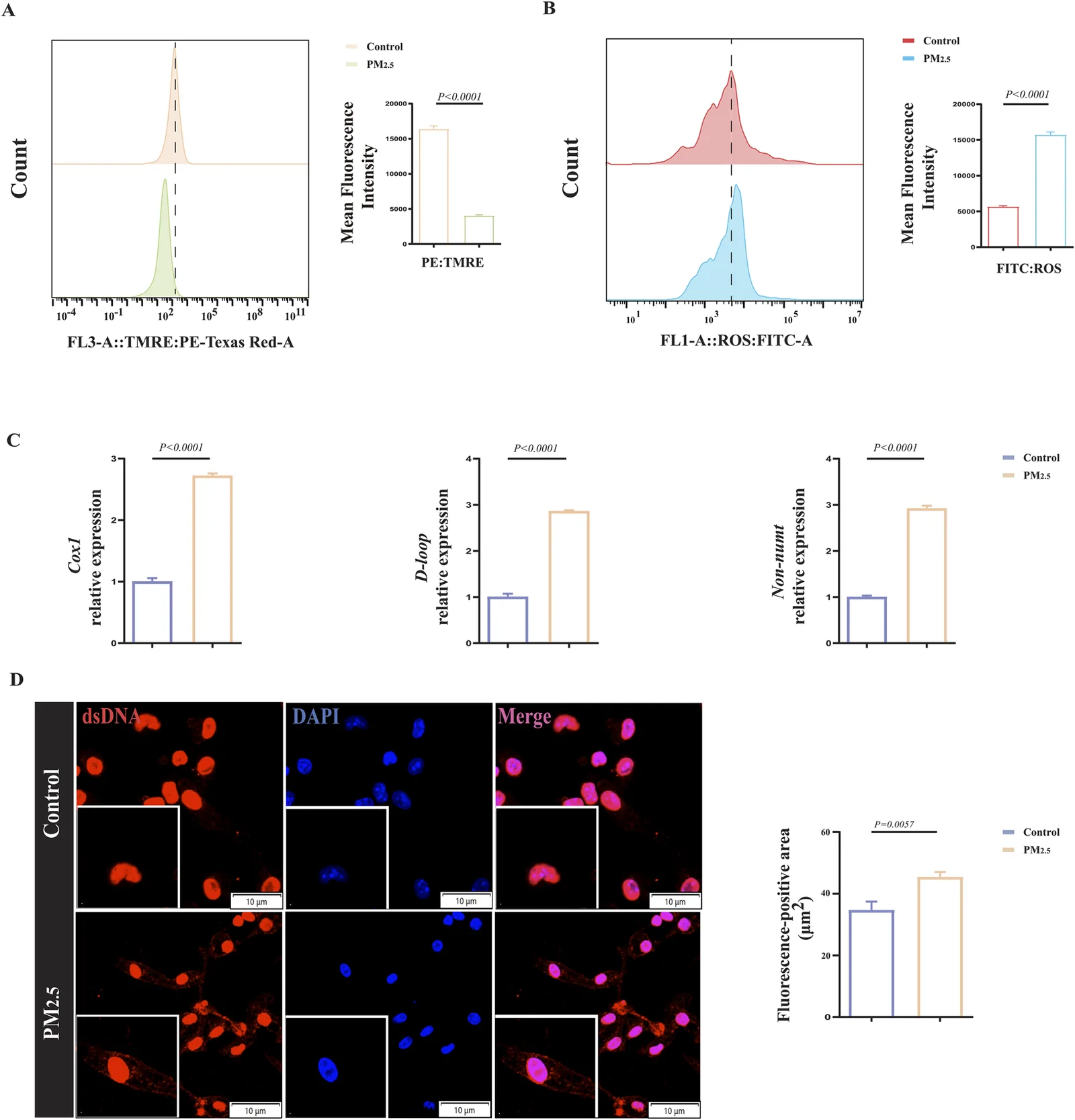
PM2.5 induced mitochondrial stress and promoted mtDNA release in macrophages. **(A,B)** Flow cytometry analysis of mitochondrial membrane potential **(A)** and ROS **(B)** in BMDMs of each group. **(C)** qRT-PCR detection of *D-loop*, *Non-numt,* and *Cox1* expression in BMDMs of each group. **(D)** Immunofluorescence staining detecting the cytoplasmic dsDNA levels in BMDM cells of each group. Data are shown as mean ± SEM, n = 3.

Mitochondria are highly susceptible to PM2.5-induced cellular stress and represent an important upstream source of innate immune activation (Fan et al., 2023). To further explore whether PM2.5-induced activation of the cGAS-STING signaling pathway in macrophages is related to mitochondrial stress, BMDMs were exposed to PM2.5 *in vitro* for verification. Flow cytometry analysis revealed that PM2.5 treatment markedly decreased mitochondrial membrane potential, as indicated by TMRE staining (Figure 5A), and significantly increased ROS levels (Figure 5B), implying that PM2.5 induced mitochondrial dysfunction and oxidative stress in macrophages. Moreover, cytosolic mtDNA of BMDM after PM2.5 exposure was detected, and qRT-PCR results showed that the levels of mtDNA-specific genes (*Cox1, D-loop, and Non-numt*) were significantly increased, suggesting that PM2.5 promoted the release of mtDNA into the cytoplasm (Figure 5C). Immunofluorescence staining revealed enhanced intracytoplasmic dsDNA signals in PM2.5-treated BMDMs (Figure 5D). The above results collectively indicate that the mitochondrial stress and mtDNA release induced by PM2.5 may serve as important upstream events contributing to cGAS-STING activation in macrophages.

### 3.6 RU.521 alleviates PM2.5-induced lung injury in mice

**FIGURE 6:**
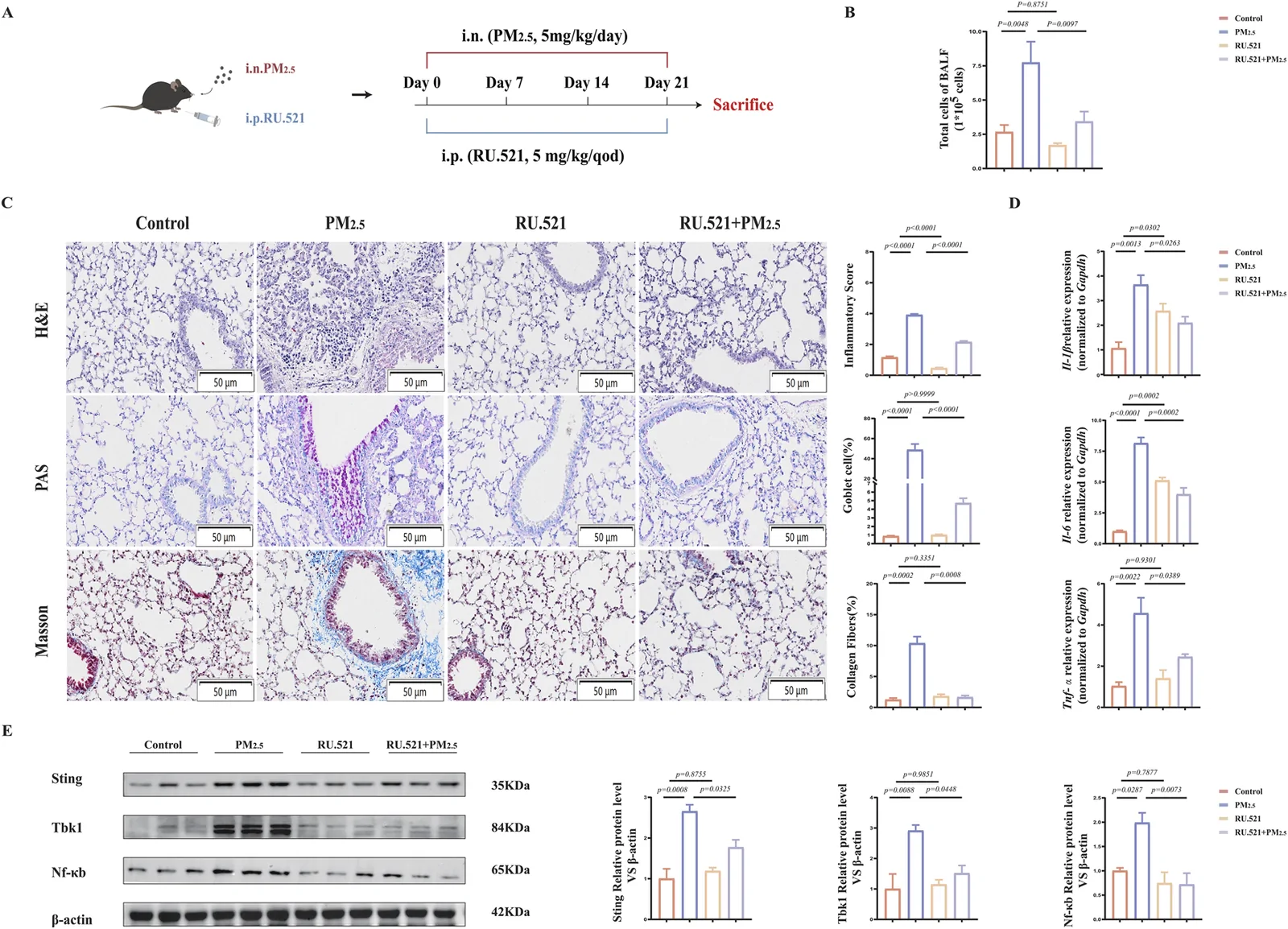
cGAS inhibitor RU.521 alleviated PM2.5-induced lung injury in mice. **(A)** Flow chart of lung injury rescue model by intranasal instillation of PM2.5 and intraperitoneal injection of RU.521. **(B)** The total cell counts in BALF of mice in each group. **(C)** H&E staining, PAS staining, and Masson staining of lung tissue in each group, as well as the inflammatory extent score of H&E staining, quantitative analysis of the positive rate of goblet cells in PAS staining, and quantitative analysis of the positive rate of collagen in Masson staining. **(D)** qRT-PCR was used to detect the expression levels of *Il-1β*, *Il-6* and *Tnf-α* in each group. **(E)** Western blot was used to analyze the protein expression levels of STING, NF-κB, and TBK1 in lung tissue of mice in each group. Data are shown as mean ± SEM, n = 3.

In order to further verify the therapeutic potential of RU.521 on lung injury induced by PM2.5 *in vivo*, a mouse model of RU.521 intervention on PM2.5 subacute exposure was established (Figure 6A). RU.521 intervention significantly reduced the total number of cells in BALF as compared with PM2.5 exposure, indicating an attenuation of pulmonary inflammatory responses (Figure 6B). H&E staining further showed that RU.521 treatment significantly reduced PM2.5-induced infiltration of inflammatory cells around the airway. Masson staining showed that PM2.5-mediated deposition of collagen fibers was reduced after RU.521 intervention, while PAS staining showed that RU.521 alleviated PM2.5-induced airway epithelial mucus secretion (Figure 6C). In addition, RU.521 significantly reduced the expression of inflammatory factors, including *Il-1β, Il-6,* and *Tnf-α* (Figure 6D), and markedly suppressed the PM2.5-induced upregulation of proteins associated with cGAS-STING signaling in lung tissues (Figure 6E). These results indicate that pharmacological inhibition of cGAS alleviated PM2.5-induced pulmonary inflammation and pathological lung injury, suggesting that cGAS-STING pathway may serve as a potential therapeutic target for PM2.5-related lung injury.

## 4 Discussion

In the present study, we demonstrated that PM2.5 subacute exposure induces significant pulmonary inflammation and tissue injury, accompanied by increased pulmonary macrophage accumulation and a shift toward the pro-inflammatory M1 phenotype. Mechanistically, PM2.5 exposure causes mitochondrial damage in macrophages, which promotes mtDNA release, activates cGAS-STING signaling, and drives M1 polarization. Pharmacological inhibition of cGAS with RU.521 suppressed macrophage M1 polarization and alleviated PM2.5-induced lung injury *in vivo*. These findings support a model in which mitochondrial stress-driven innate immune sensing amplifies macrophage inflammatory polarization and thereby contributes to PM2.5-induced lung injury.

Macrophages are central regulators of pulmonary innate immunity and are essential for maintaining tissue homeostasis under physiological conditions. Previous studies have demonstrated that M1 macrophages produced proinflammatory cytokines and chemokines to amplify local inflammation and worsen tissue damage (Lu et al., 2018). Our results reveal that PM2.5 exposure significantly increased macrophage accumulation in lung tissue and promoted polarization toward the M1 phenotype, as evidenced by elevated expression of pro-inflammatory cytokines and chemokines along with reduced expression of M2-associated markers. These findings indicated that PM2.5 drove macrophages toward an inflammatory M1 phenotype, creating a proinflammatory microenvironment. Such polarization promoted sustained release of pro-inflammatory mediators and inflammatory cell infiltration, forming a positive feedback loop that aggravated tissue injury and delayed inflammation resolution. Notably, endotoxin, mainly represented by lipopolysaccharide (LPS), is a well-established inducer of M1 macrophage polarization (Wu et al., 2026). Previous studies have reported that LPS can be present in ambient PM2.5 preparations (Shen et al., 2024), suggesting that endotoxin contamination may partially contribute to PM2.5-induced macrophage activation. In the present study, endotoxin levels in the PM2.5 preparation were not directly measured; therefore, the potential contribution of endotoxin to macrophage M1 polarization cannot be completely excluded. Future studies using endotoxin-tested PM2.5 preparations or endotoxin-removal strategies are warranted to further clarify the specificity of the PM2.5-induced macrophage polarization mechanism.

In recent years, studies have shown that the role of innate immune signaling pathways in sterile inflammation and diseases associated with environmental exposure has attracted increasing attention. As an important hub for cytoplasmic DNA sensing, cGAS-STING not only mediates the production of type I interferons and proinflammatory factors in anti-infective immunity, but also participates in sterile inflammatory processes such as ischemia-reperfusion injury and autoimmunity by recognizing self-derived DNA (Geng et al., 2023). Our results indicated that PM2.5 activated the cGAS-STING signaling pathway in macrophages, thereby driving inflammatory responses. Further pharmacological intervention confirmed that the cGAS inhibitor RU.521 markedly attenuated PM2.5-induced macrophage inflammatory polarization, proinflammatory cytokine release, and inflammatory lung injury. These findings highlight the central role of cGAS-STING signaling pathway in linking environmental stress to macrophage-mediated inflammation.

Mitochondria are not only central to cellular energy metabolism but also play a crucial role in regulating innate immune responses. Under various stress conditions or cellular injury, mitochondrial dysfunction can lead to excessive production of reactive oxygen species (ROS), significant reduction in membrane potential, and increased membrane permeability, thereby promoting the release of mitochondrial DNA (mtDNA) into the cytoplasm. As a damage-associated molecular pattern (DAMP) with structural similarities to bacterial DNA, mtDNA can be recognized by cytoplasmic DNA-sensing pathways, which further activates the cGAS–STING signaling pathway and triggers downstream inflammatory responses (Leclercq et al., 2018; Chen et al., 2023). Meanwhile, our current findings confirm that PM2.5 exposure induced mitochondrial damage and promoted mtDNA release into the cytoplasm. Combining previous research findings, we propose that PM2.5 induces mitochondrial dysfunction, which in turn leads to mtDNA release and activates the cGAS–STING pathway. Thus, mitochondrial injury and mtDNA release are key upstream triggers for PM2.5-induced cGAS-STING activation and lung injury.

**FIGURE 7:**
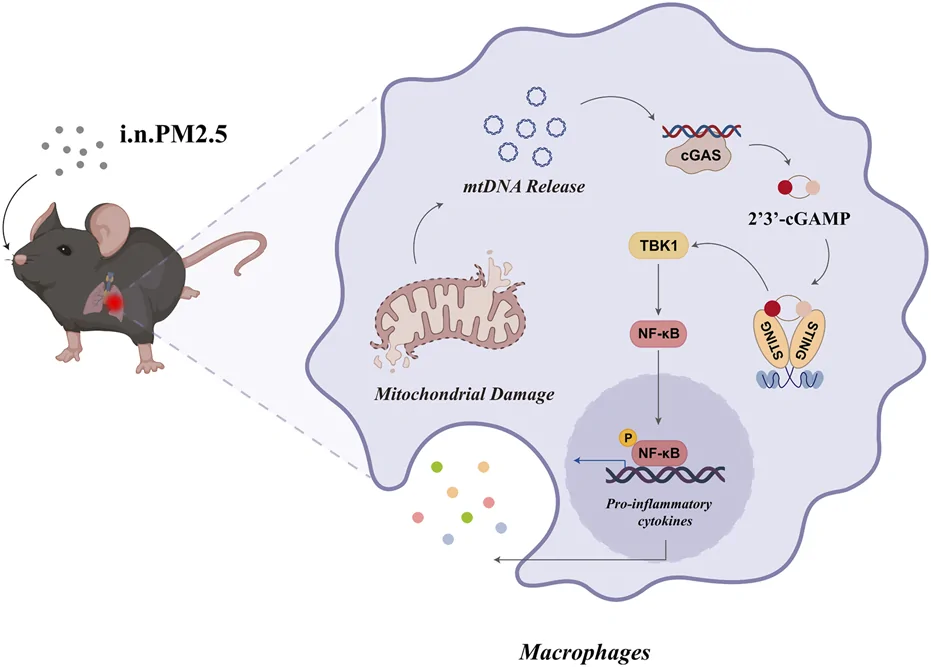
Schematic representation of PM2.5-induced macrophage M1 polarization via the mtDNA-cGAS-STING axis. PM2.5-induced mitochondrial damage in macrophages promotes cytosolic mtDNA release, which activates the cGAS-STING pathway and downstream TBK1/NF-κB signaling. NF-κB activation drives the release of pro-inflammatory cytokines, thereby promoting macrophage polarization toward the pro-inflammatory M1 phenotype and amplifying pulmonary inflammation.

In conclusion, our study supports a model in which PM2.5 subacute exposure induces mitochondrial stress and mtDNA release into the cytosol in macrophages, thereby activating cGAS-STING signaling, promoting M1 polarization, and amplifying pulmonary inflammation and tissue remodeling (Figure 7). These findings identify macrophage cGAS-STING activation as a key link between mitochondrial dysfunction and inflammatory lung injury, providing an integrated framework for understanding PM2.5-induced immunopathology.

## Data Availability

The original contributions presented in the study are included in the article/Supplementary Material, further inquiries can be directed to the corresponding authors.
