# Supplementary material for: PM2.5 induces lung injury via mtDNA-cGAS-STING-mediated macrophage M1 polarization
Source: Front Pharmacol. 2026 Aug 6;17:1902928. doi: 10.3389/fphar.2026.1902928 (PMC13489883; doi:10.3389/fphar.2026.1902928)
Supplement: Supplementary file 1 [file Table1.doc]

| **Gene name** | Primer sequence（5’ – 3’） |
| --- | --- |
| *Gapdh* | F：AGGTCGGTGTGAACGGATTTG |
|  | R：TGTAGACCATGTAGTTGAGGTCA |
| *Il-1β* | F：GCAACTGTTCCTGAACTCAACT |
|  | R：ATCTTTTGGGGTCCGTCAACT |
| *Il-6* | F：TAGTCCTTCCTACCCCAATTTCC |
|  | R：TTGGTCCTTAGCCACTCCTTC |
| *Il-10* | F：GCTCTTACTGACTGGCATGAG |
|  | R：CGCAGCTCTAGGAGCATGTG |
| *Tnf-α* | F：CCCTCACACTCAGATCATCTTCT |
|  | R：GCTACGACGTGGGCTACAG |
| *iNos* | F：GTTCTCAGCCCAACAATACAAGA |
|  | R：GTGGACGGGTCGATGTCAC |
| *Arg1* | F：ATGCTCACACTGACATCAAC |
|  | R：TCTCTTCCATCACCTTGCC |
| *Cgas* | F：GAGGCGCGGAAAGTCGTAA |
|  | R：TTGTCCGGTTCCTTCCTGGA |
| *Sting* | F：GGTCACCGCTCCAAATATGTAG |
|  | R：CAGTAGTCCAAGTTCGTGCGA |
| *Tbk1* | F：ACTGGTGATCTCTATGCTGTCA |
|  | R：TTCTGGAAGTCCATACGCATTG |
| *Irf3* | F：GAGAGCCGAACGAGGTTCAG |
|  | R：CTTCCAGGTTGACACGTCCG |
| *Nfkb* | F：ATGGCAGACGATGATCCCTAC |
|  | R：TGTTGACAGTGGTATTTCTGGTG |
| *Cxcl1* | F：ACTCAAGAATGGTCGCGAGG |
|  | R：GTGCCATCAGAGCAGTCTGT |
| *Cxcl2*  *D-loop*  *Non-numt*  *Cox1*  *Tert* | F：GCGCCCAGACAGAAGTCATA |
|  | R：CGAGGCACATCAGGTACGAT  F：AATCTACCATCCTCCGTGAAACC  R：TCAGTTTAGCTACCCCCAAGTTTAA  F：CTAGAAACCCCGAAACCAAA  R：CCAGCTATCACCAAGCTCGT  F：GCCCCAGATATAGCATTCCC  R：GTTCATCCTGTTCCTGCTCC  F：CTAGCTCATGTGTCAAGACCCTCTT  R：GCCAGCACGTTTCTCTCGTT |

Table S1. Primer sequences used for RT-qPCR.
